# Coexisting *BRAF*-Mutated Langerhans Cell Histiocytosis and Primary Myelofibrosis with Shared *JAK2* Mutation

**DOI:** 10.1155/2021/6623706

**Published:** 2021-04-16

**Authors:** Johanne Marie Holst, Marie Beck Enemark, Trine Lindhardt Plesner, Martin Bjerregaard Pedersen, Maja Ludvigsen, Francesco d'Amore

**Affiliations:** ^1^Department of Hematology, Aarhus University Hospital, Palle Juul-Jensens Boulevard 35, Aarhus N 8200, Denmark; ^2^Department of Clinical Medicine, Aarhus University, Palle Juul-Jensens Boulevard 82, Aarhus N 8200, Denmark; ^3^Department of Pathology, Copenhagen University Hospital, Blegdamsvej 3B, København N 2200, Denmark

## Abstract

Langerhans cell histiocytosis (LCH) is an infrequent disease, characterized by oligoclonal proliferation of immature myeloid-derived cells. However, the exact pathogenesis remains unknown. In rare cases, LCH is present in patients with concomitant myeloid proliferative neoplasms. Here, we describe a 69-year-old male, who presented with a maculopapular rash covering truncus, face, and scalp. A cutaneous ulcerating lesion on the right cheek led to a biopsy showing LCH. Lesional cells were *BRAF*^V600E^ and *JAK2*^V617F^ mutated. A bone marrow aspirate showed no infiltration of Langerhans cells, but alterations consistent with primary myelofibrosis (PMF) and a polymerase chain reaction test were positive for *JAK2*^V617F^. Our case highlights an uncommon condition of two hematological malignancies present in the same patient. The identification of the *BRAF*^V600E^ mutation supports previous findings of this mutation in LCH. Interestingly, a *JAK2*^V617F^ mutation was found in both LCH and PMF cells, indicating a possible clonal relationship between the two malignancies.

## 1. Introduction

Langerhans cell histiocytosis (LCH) is a clonal neoplastic proliferation of immature myeloid-derived cells similar to Langerhans cells [1–3]. The disease is rare and affects adults at a rate of approximately 1-2 cases per million [3, 4]. It may present as localized to a single site or occur as more disseminated disease with multiorgan involvement. The clinical course is highly related to disease stage and type of organ involvement with high survival rates in unifocal disease as opposed to multisystem disease, particularly if resistant to therapy [1]. The pathogenesis of LCH is poorly understood, but the neoplastic cells are believed to originate from immature myeloid-derived dendritic cells, similar to epidermal Langerhans cells [1, 3]. Approximately 50% of LCH cases harbor *BRAF*^V600E^ mutations in LCH cells resulting in constitutive Ras/Raf/MEK/ERK signaling and increased mitogen-activated protein kinase (MAPK) activity [4–7]. LCH has previously been reported associated with other hematological disorders, including cases of myeloid proliferative neoplasia (MPN) [8, 9]. Here, we present a rare case presentation of *BRAF*^V600E^-mutated LCH and coexisting primary myelofibrosis (PMF) both sharing a Janus kinase 2 (*JAK2*)^V617F^ mutation.

## 2. Case Presentation

**Figure 1:**
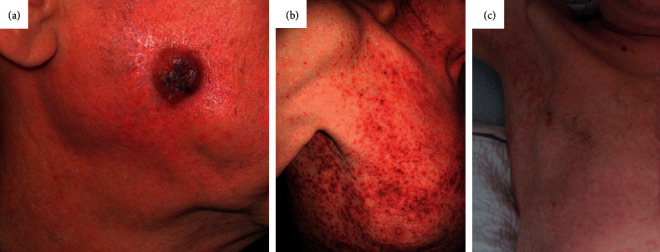
(a) Tumor localized to the cheek of the patient. (b) Painless, nonitching maculopapular rash on the patient's truncus. (c) Normalization of the skin after BRAF-inhibitor treatment.

**Figure 2:**
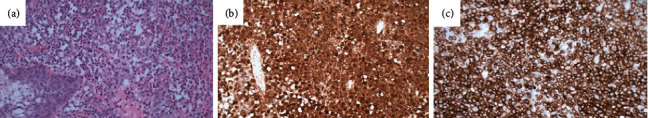
Skin sample at the time of diagnosis showing histopathological and immunohistochemical features consistent with Langerhans cell histiocytosis. (a) Haematoxylin and eosin staining (100x), and immunohistochemical staining for (b) S100 (100x) and (c) CD1a (100x).

A 69-year-old man was admitted to our department after skin biopsies from the ear and right cheek (Figure 1(a)) had revealed a histopathological picture morphologically and immunohistochemically consistent with LCH (Figure 2). Due to a painless, nonitching maculo-papular rash on face, scalp, and truncus (Figure 1(b)), he was treated with phototherapy (i.e., UVB) and topical steroids by a local dermatologist for approximately one year. During the last three months prior to admission, a 2.5 cm × 2.5 cm ulcerative cutaneous lesion had developed on his cheek. He had symptomatic generalized pruritus and fatigue but no night sweats, weight loss, or bone pain. Neither peripheral lymph adenopathy nor hepatosplenomegaly were found. Blood counts showed mild anemia with hemoglobin of 12.9 g/dL, a total leucocyte count of 14.5 × 10^9^/L, and platelets of 451 × 10^9^/L. During the following year, the patient continued to have anemia, sustained leukocytosis, and a varying level of thrombocytes. At diagnosis, serum-bilirubin, serum-aminotransferase, serum-lactate dehydrogenase, and serum-creatinine levels were normal.

Immunohistochemically, a biopsy from the skin and ear showed a tumor cell population with a strong expression of S100 and CD1a (CD207) and moderate expression of CD68 (Figures 2(b) and 2(c)). The proliferation rate of the neoplastic cells was 40% (Ki-67). Both *BRAF*^V600E^ mutation and *JAK2*^V617F^ mutation (variant allele frequency (VAF) 0.08%) were detected in the sample using a quantitative polymerase chain reaction (qPCR) assay with a sensitivity of 1% and 0.01%, respectively.

**Figure 3:**
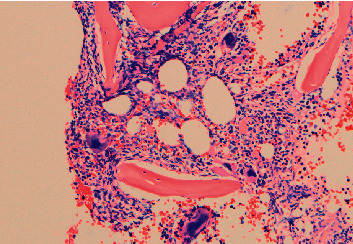
Giemsa staining of the bone marrow sample showing histopathological features consistent with primary myelofibrosis (200x).

A trephine biopsy showed no evidence of LCH in the bone marrow, but the histomorphology was consistent with a chronic myeloproliferative neoplasia of PMF type (Figure 3). The bone marrow was hypercellular with an elevated number of clustered atypic megakaryocytes (large hyperlobulated in varying shapes and with large hyperchromatic nuclei). Increased reticulin and early-stage collagen fibrosis were seen. A *JAK2*^V617F^ mutation (VAF 0.2%) was identified, while other common MPN-associated mutations in genes such as *CALR *or *MPL* were not found. No *BRAF* mutation was identified in the bone marrow.

**Figure 4:**
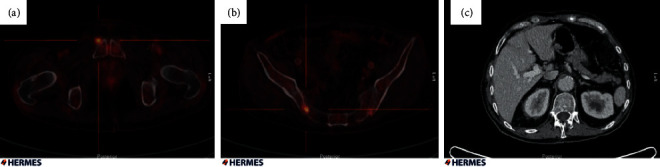
Elevated ^18^F-FDG uptake localized corresponding to (a) the right cheek, cutaneous portion of the back head (occipital) and in one lymph node at the neck and (b) several foci in the pelvic bones, and a soft tissue process ahead of right os pubis showing increased ^18^F-FDG activities. (c) The CT scan showing pathological edematous appearance of the kidney surrounded by adipose tissue.

In order to determine the extent of disease dissemination, a combined positron emission tomography-computed tomography (PET/CT) scan was performed. It revealed increased focal ^18^F-FDG uptake localized to the right cheek lesion, a cutaneous lesion in the occipital area, a cervical lymph node, the soft tissue anterior to the pubic bone (Figure 4(a)), and several foci in pelvic bones (Figure 4(b)). Thus, the patient suffered from multifocal LCH. The CT scan revealed an edematous appearance of both kidneys surrounded by adipose tissue similar to the appearance described in patients with Erdheim–Chester disease (Figure 3(c)). However, a kidney biopsy showed no infiltration of LCH.

The patient had a medical history of multiple cardiovascular events on an atherosclerotic background including coronary bypass grafting, bypass surgery of the leg, and a cerebral insult with moderate cognitive sequelae. His medication consisted of tamsulosin, clopidogrel, and simvastatin. He was a nonsmoker and had no history of alcohol abuse.

His family history included several cases of cancer, including a brother with chronic lymphoid leukemia and a niece treated for acute lymphocytic leukemia.

The patient was treated with IV cytarabine (100 mg/m^2^), which had to be prematurely discontinued due to infectious complications. The patient was mainly affected by the LCH skin alterations, and as the disease progressed, subsequently, the patient was treated with the oral BRAF-inhibitor vemurafenib for three months. This considerably improved his skin alterations (Figure 1(c)). Nine treatments of topical application of mechlorethamine were performed in the same period. Following a planned lower-extremity thrombectomy, the patient developed an acute coronary infarction. Sadly, he died of infectious complications one year after the initial diagnosis of LCH and PMF.

## 3. Discussion

Here, we describe the clinical, histopathological, and molecular findings in a patient who was diagnosed with two rare coexisting cancers of hematopoietic origin, i.e., LCH and PMF. While a *BRAF* mutation seemed limited to the LCH-associated lesions, a *JAK2*^V617F^ mutation was found in both the cutaneous LCH lesion and in the LCH-void, but PMF involved bone marrow. The latter observation may suggest a possible pathogenetic relationship between the two malignant conditions in this patient. To our knowledge, the occurrence of coexisting LCH and PMF has only been described once before [10]. In that report, LCH and PMF were demonstrated to share the *JAK2*^V617F^ mutation by performing a molecular genetic analysis on laser microdissected single LCH and PMF cells, suggesting a possible clonal relationship between the two cell populations.

LCH is characterized by proliferating clonal neoplastic cells with surface markers similar to those of normal Langerhans cells found in the skin [1–3]. However, the origin of the malignant cell population in LCH has been questioned [11], and a comparison of global gene and protein expression patterns between LCH cells and epidermal Langerhans cells shows surprisingly low overall correlation [12]. Furthermore, numerous of the overexpressed genes in LCH cells are known to be associated with myeloid-derived dendritic cells. Some previous reports have found LCH associated with other hematopoietic disorders, including myelo- and lymphoproliferative malignancies [8, 9, 13, 14]. It has therefore been hypothesized that mutations in common hematopoietic precursor cells may explain the development of occasional co-occurrence of two hematological diseases [8, 15]. Interestingly, Milne et al. found *BRAF*^V600E^ mutations present both in myeloid progenitors and CD34^+^ cells from LCH patients [16]. Our finding of a shared *JAK2* mutation in the LCH and PMF lesions of our patient supports the hypothesis of common pathogenetic steps, possibly at the hematopoietic progenitor cell level, leading to the development of both diseases. Both *BRAF* and *JAK2* are genes whose regulatory impact lies upstream of the MAPK and Pi3K pathways [5, 17]. Hence, the occurrence of a shared *JAK2* mutation may suggest a concurrent development of LCH and PMF from clonally related hematopoietic progenitor cell populations.

The qPCR was performed on DNA extracted from bone marrow and skin biopsies with a substantial contribution from nonneoplastic cells. This may in part explain the low allelic frequencies of *JAK2* mutations identified in the samples. Presence of blood cells in the LCH skin biopsy resulting in identification of the same *JAK2* mutation in the LCH skin biopsy may therefore also represent a possible pitfall.

Both LCH and PMF are associated with chronic inflammation, and this may contribute to the pathophysiology in both diseases [1, 18–20]. With this case, we show interesting possible insight into the pathogenesis of LCH with the association of both the MAPK and JAK-STAT pathways, which are both involved in various immunologic and inflammatory responses. Furthermore, the identification of *BRAF*^V600E^ and constitutive MAPK and Ras/Raf/MEK/ERK signaling has contributed to the establishment of LCH as a malignant process [4, 21]. BRAF signaling is involved in various cellular functions, such as proliferation, apoptosis, angiogenesis, and survival. Accordingly, *BRAF* mutations have been associated with various cancers, e.g., hairy cell leukemia and metastatic melanoma [4, 22, 23]. The *BRAF* enzyme inhibitor vemurafenib represents an attractive novel treatment option in *BRAF*^V600E^-mutated cancers [24]. Also, our patient had a remarkable clinical benefit from the treatment with vemurafenib leading to considerable improvement of the original facial skin alterations. The short observation period on vemurafenib treatment due to the patient's death following complications after a planned vascular surgery procedure does not allow us to evaluate whether vemurafenib also was beneficial on PMF.

## 4. Conclusion

In conclusion, here we describe a patient with coexisting *BRAF*^V600E^-mutated LCH and PMF sharing a *JAK2*^V617F^ mutation. Whether a possible molecular relationship between LCH and MPN exists—and if the diseases, in some cases, share common pathologic driving events—will require further investigations on a larger number of cases, and, if possible, investigated with careful single-cell analysis.

## Data Availability

Original clinical pathological data are obtained from the patient's medical record with written informed consent as well as approval from the Danish Data Protection Agency (record no. 1-16-02-420-15) and the Danish National Committee on Health Research Ethics (record no. 1609521).
